# Epidemiology of Coronavirus Disease Outbreak among Crewmembers on Cruise Ship, Nagasaki City, Japan, April 2020

**DOI:** 10.3201/eid2709.204596

**Published:** 2021-09

**Authors:** Haruka Maeda, Eiichiro Sando, Michiko Toizumi, Yuzo Arima, Tomoe Shimada, Takeshi Tanaka, Masato Tashiro, Ayumi Fujita, Katsunori Yanagihara, Hayato Takayama, Ikkoh Yasuda, Nobuyuki Kawachi, Yoshitaka Kohayagawa, Maiko Hasegawa, Katsuaki Motomura, Rie Fujita, Katsumi Nakata, Jiro Yasuda, Koichi Morita, Shigeru Kohno, Koichi Izumikawa, Motoi Suzuki, Konosuke Morimoto

**Affiliations:** Nagasaki University, Nagasaki, Japan (H. Maeda, E. Sando, M. Toizumi, T. Tanaka, M. Tashiro, A. Fujita, K. Yanagihara, H. Takayama, I. Yasuda, N. Kawachi, J. Yasuda, K. Morita, S. Kohno, K. Izumikawa, K. Morimoto);; Fukushima Medical University, Fukushima, Japan (E. Sando, I. Yasuda);; National Institute of Infectious Diseases, Tokyo, Japan (Y. Arima, T. Shimada, M. Suzuki);; Disaster Medical Center of Japan, Tokyo (Y. Kohayagawa);; Nagasaki Prefecture Government, Nagasaki (M. Hasegawa, K. Nakata);; Nagasaki City Public Health Center, Nagasaki (K. Motomura);; Northern Nagasaki Public Health Center, Nagasaki (R. Fujita)

**Keywords:** COVID-19, coronavirus disease, SARS-CoV-2, severe acute respiratory syndrome coronavirus 2, viruses, respiratory infections, zoonoses, outbreak, cruise ship, Japan, epidemiology

## Abstract

In April 2020, a coronavirus disease (COVID-19) outbreak occurred on the cruise ship Costa Atlantica in Nagasaki, Japan. Our outbreak investigation included 623 multinational crewmembers onboard on April 20. Median age was 31 years; 84% were men. Each crewmember was isolated or quarantined in a single room inside the ship, and monitoring of health status was supported by a remote health monitoring system. Crewmembers with more severe illness were hospitalized. The investigation found that the outbreak started in late March and peaked in late April, resulting in 149 laboratory-confirmed and 107 probable cases of infection with severe acute respiratory syndrome coronavirus 2. Six case-patients were hospitalized for COVID-19 pneumonia, including 1 in severe condition and 2 who required oxygen administration, but no deaths occurred. Although the virus can spread rapidly on a cruise ship, we describe how prompt isolation and quarantine combined with a sensitive syndromic surveillance system can control a COVID-19 outbreak.

Severe acute respiratory syndrome coronavirus 2 (SARS-CoV-2) was first reported from Wuhan, China (*1*), and led to outbreaks of coronavirus disease (COVID-19), which was declared a pandemic by the World Health Organization on March 11, 2020. COVID-19 also has been affecting global economies, leading to several recessions (*2*). Japan experienced an outbreak of COVID-19 on the cruise ship Diamond Princess during the early stages of the epidemic in February 2020 (*3*–*5*). The government of Japan prohibited entry into the country at the end of March, declaring a state of emergency in 7 prefectures on April 7, which became a nationwide policy on April 16. Against this backdrop, the Italian cruise ship Costa Atlantica had remained docked at Nagasaki City since January 2020 for full maintenance. In April 2020, we identified an outbreak of COVID-19 on this cruise ship.

COVID-19 spreads easily on cruise ships because of the “3 Cs”: crowded places, close-contact settings, and confined and enclosed spaces (*6*–*9*). Given the specialized setting of a cruise ship and its closed population, a cruise ship can offer important insights about infectious disease epidemiology and transmission dynamics (*10*). How to manage an outbreak of COVID-19 on a cruise ship is a matter of debate, especially in a resource-limited situation. To improve our understanding of COVID-19 and prepare for outbreaks to come, studies of outbreaks on cruise ships are valuable. In this article we describe the epidemiology of the COVID-19 outbreak on Costa Atlantica and approaches taken for managing and responding to this outbreak.

## Methods

### Setting

On April 19, 2020, officials of Costa Atlantica, which had been docked in Nagasaki City since January 2020, reported to Nagasaki City Public Health Center that they had febrile crewmembers (*11*,*12*). No passengers were on board the ship. All 623 crewmembers had already completed their quarantine upon entry in Japan, but they had been asked to refrain from leaving the ship unless necessary as part of the public health policy to prevent the spread of COVID-19. Because of cruise ship employment contracts, 56 crewmembers embarked during March 14–April 3. Given the COVID-19 pandemic, the body temperature of all crewmembers had been checked daily since the end of February. Beginning March 22, at the discretion of the cruise ship company, any crewmember with a body temperature >37.1°C was to be isolated in a single-passenger cabin room of the ship; beginning April 19, every nonessential worker was isolated or quarantined in a single-passenger cabin room. Essential workers were defined as crewmembers who were involved in the operation of the ship or in maintaining its operation and functionality, such as the captain, engineers, and food preparation staff.

On April 20, we performed PCR assays for SARS-CoV-2 for 4 crewmembers who had a body temperature >37.1°C, resulting in 1 positive result. During April 21–25, all crewmembers underwent universal screening for infection by using loop-mediated isothermal amplification (LAMP) for SARS-CoV-2. After the universal screening, each nonessential worker remained isolated or quarantined in a single passenger cabin room. Even for those whose test results were positive, crewmembers with mild illness or without signs or symptoms remained on the ship, and the health status of all crewmembers was monitored daily. If clinically indicated, regardless of test results, ill persons were transported and admitted to hospitals in Nagasaki City at the discretion of the ship’s medical doctor.

### Data Collection and Definitions

The study population included 623 crewmembers who were on board on April 20, 2020. The cruise ship company provided demographic and body temperature data (ship’s medical record) during March 14–May 27 for all crewmembers on board. Demographic data included sex, date of birth, nationality, and occupation category. Before disembarkation from the ship, crewmembers also provided information regarding their smoking history, presence of any underlying disease, height and weight, daily body temperature, and clinical signs or symptoms during April 28–May 29 by using a smartphone-based remote health monitoring system (*13*). Obesity, as a risk factor for severe COVID-19, was defined as a body mass index (BMI) >30 (*14*,*15*). Clinical signs and symptoms of COVID-19 were fever (body temperature >37.5°C), cough, shortness of breath, nasal congestion, sore throat, nausea or vomiting, conjunctival congestion, headache, fatigue, myalgia or arthralgia, diarrhea, olfactory dysfunction, and taste disorder (loss of taste), which are globally recognized COVID-19 signs and symptoms (*1*,*13*,*16*–*18*). In managing this outbreak, the threshold value of a body temperature >37.1°C was applied on the basis of the cruise ship’s definition for illness and criteria for isolation precaution.

We defined laboratory-confirmed cases as illness in anyone with a positive test result for SARS-CoV-2 by PCR or LAMP. We defined a probable case was defined as illness in anyone with signs or symptoms indicative of COVID-19 but with a negative test result (*19*). We divided the severity of COVID-19 into 4 groups (*20*): severe pneumonia that required intubation or intensive care unit admission, moderate pneumonia that required oxygen administration, mild illness with COVID-19 signs or symptoms that did not require oxygen administration, and an asymptomatic condition without any clinical signs or symptoms. We performed chest radiographs or chest computed tomography scans only for those suspected of having pneumonia, such as prolonged fever or shortness of breath, and those who were hospitalized.

### Testing Strategy

We confirmed SARS-CoV-2 infection by using PCR or LAMP. We conducted PCR according to the protocol recommended by Japan’s National Institute of Infectious Diseases (*21*). LAMP is used for the detection of SARS-CoV-2 because of its fast turnaround time and acceptable levels of sensitivity and specificity (*22*–*24*). LAMP was conducted at the Institute of Tropical Medicine at Nagasaki University and Nagasaki University Hospital. Persons who tested positive were allowed to disembark and travel back to their countries after negative test results were confirmed in subsequent tests and their signs or symptoms had resolved.

### Data Analysis

We constructed an epidemic curve on the basis of illness onset date, which was based on a body temperature >37.1°C according to the ship’s medical record or the smartphone-based health monitoring system; the onset date of body temperature >37.1°C was defined as the date when body temperature was >37.1°C with a body temperature <37.1°C until the previous day. For the epidemic curve, we used body temperature >37.1°C for 2 reasons: first, it was the cruise ship’s definition for illness and criteria for isolation precaution; second, describing the epidemic curve based on the test results of SARS-CoV-2 from the universal screening would not give an accurate picture of this outbreak because screening was introduced ≈1 month after the beginning of the outbreak and would only detect infections prevalent at the time of screening.

In addition to monitoring such incident events, we monitored the daily prevalent numbers of crewmembers with a body temperature >37.1°C or signs or symptoms. To evaluate the spatial distribution of infection, we plotted on the ship’s map the cabin rooms of crewmembers with a body temperature >37.1°C before isolation. We noted the demographic characteristics of all crewmembers according to their test results and presence of signs or symptoms. We calculated attack rates for both laboratory-confirmed case-patients (laboratory-confirmed case-patients divided by all crewmembers) and with the addition of probable case-patients (laboratory-confirmed case-patients and probable case-patients divided by all crewmembers). We also plotted the clinical course of symptomatic crewmembers individually by calendar date.

We expressed continuous variables as medians and interquartile ranges (IQRs). We summarized categoric variables as numbers and proportions. We conducted statistical analyses by using Stata 16 (StataCorp, https://www.stata.com).

### Ethics

The governments of Nagasaki City and Nagasaki Prefecture conducted the surveillance of the COVID-19 outbreak on this cruise ship during April 20–May 29, 2020, under authorization by the Infectious Diseases Control Law. This study was approved by the Institutional Review Board at the Institute of Tropical Medicine at Nagasaki University (approval no. 200619242).

## Results

### Demographic Characteristics of the Crewmembers

Among the 623 crewmembers, the median age was 31 years (IQR 26–40 years), and 84% (523/623) were men (Table 1). Thirty-six nations were represented by the crewmembers, including (in descending order) the Philippines, India, Indonesia, and China; most (80%) crewmembers were from countries in Asia. Characteristic data were available for 593 crewmembers. Of those, 25% (148/592) had a history of smoking, and 3.7% (22/593) had underlying diseases, including hypertension (2.0% [12/592]), diabetes (1.7% [10/592]), cardiovascular disease (0.2% [1/592]), and asthma (0.2% [1/592]). Median BMI was 24.1 (IQR 21.7–26.7), and 9.4% (49/523) crewmembers had obesity (BMI >30).

**Table 1:** Selected characteristics of crewmembers on cruise ship where a coronavirus disease outbreak occurred, by SARS-CoV-2 test result and symptomatic status, Nagasaki, Japan, 2020*

Characteristic	All crewmembers	Test-positive, n = 149			Test-negative, n = 474	
		Symptomatic	Asymptomatic		Symptomatic	Asymptomatic
Total	623 (100)	96 (100)	53 (100)		107 (100)	367 (100)
Age, y						
Median (IQR)	31 (26–40)	32 (27–39)	32 (23–42)		29 (25–35)	31 (26–41)
Distribution						
10–19	3 (0.5)	1 (1.0)	1 (1.9)		0	1 (0)
20–29	271 (43)	37 (39)	20 (38)		55 (51)	159 (43)
30–39	184 (30)	36 (38)	14 (26)		33 (31)	101 (28)
40–49	123 (20)	17 (18)	15 (28)		13 (12)	78 (21)
50–59	34 (5.5)	4 (4.2)	2 (3.8)		6 (5.6)	22 (6.0)
>60	8 (1.3)	1 (1.0)	1 (1.9)		0	6 (1.6)
Sex						
M	523 (84)	82 (85)	46 (87)		88 (82)	307 (84)
F	100 (16)	14 (15)	7 (13)		19 (18)	60 (16)
Nationality						
Philippines	206 (33)	35 (36)	6 (11)		40 (37)	125 (34)
India	104 (17)	16 (17)	14 (26)		16 (15)	58 (16)
Indonesia	84 (13)	10 (10)	13 (25)		13 (12)	48 (13)
China	82 (13)	14 (15)	7 (13)		13 (12)	48 (13)
Italy	40 (6.4)	5 (5.2)	3 (5.7)		6 (5.6)	26 (7.1)
Other	107 (17)	16 (17)	10 (19)		19 (18)	62 (17)
Occupation category						
Essential worker						
Engine	75 (12)	6 (6.3)	11 (21)		10 (8.1)	48 (13)
Hotel	73 (12)	13 (14)	3 (5.7)		6 (5.0)	51 (14)
Deck	70 (11)	11 (11)	10 (19)		11 (9.4)	38 (10)
Nonessential worker						
Restaurant	96 (15)	13 (14)	6 (11)		21 (20)	56 (15)
Galley	77 (12)	18 (19)	5 (9.4)		22 (21)	32 (8.7)
Housekeeping	72 (12)	8 (8.3)	7 (13)		7 (6.5)	50 (14)
Entertainment	49 (7.9)	8 (8.3)	6 (11)		13 (12)	22 (6.0)
Technician	21 (3.4)	4 (4.2)	2 (3.8)		3 (2.8)	12 (3.3)
Others	90 (14)	15 (16)	3 (5.7)		14 (13)	58 (16)
Smoking history, n = 592†						
Yes	148 (25)	20 (22)	13 (28)		36 (35)	79 (23)
Underlying disease, n = 593†‡						
Any	22 (3.7)	5 (5.5)	2 (4.4)		2 (1.9)	13 (3.7)
Hypertension	12 (3.0)	2 (2.2)	1 (2.2)		0	9 (2.6)
Diabetes	10 (1.7)	1 (1.1)	1 (2.2)		0	8 (2.3)
Cardiovascular disease	1 (0.2)	1 (1.1)	0		0	0
Asthma	1 (0.2)	0	0		1 (1.0)	0
BMI, n = 523†						
Median (IQR)	24.1 (21.7–26.7)	23.6 (21.3–26.0)	24.7 (21.6–28.0)		23.9 (21.3–26.7)	24.1 (22.0–26.5)
Distribution						
Underweight, BMI < 18.5	29 (5.5)	5 (6.2)	3 (7.5)		2 (2.0)	19 (6.3)
Normal, 18.5 < BMI < 25	278 (53)	45 (56)	18 (45)		58 (59)	157 (52)
Overweight, 25 < BMI < 30	167 (32)	22 (27)	15 (36)		27 (28)	103 (34)
Obese, BMI >30	49 (9.4)	9 (11)	4 (10)		11 (11)	25 (8.2)

*Values are no. (%) except as indicated. Symptomatic was defined as having any clinical sign or symptom of coronavirus disease (i.e., fever (>37.5°C), cough, shortness of breath, nasal congestion, sore throat, nausea or vomiting, conjunctival congestion, headache, fatigue, myalgia or arthralgia, diarrhea, olfactory dysfunction, or taste disorder [loss of taste]). BMI, body mass index; IQR, interquartile range, SARS-CoV-2, severe acute respiratory coronavirus 2. †Among crewmembers who entered data into the health monitoring system since its introduction on April 28, 2020. ‡Two crewmembers had both hypertension and diabetes.

### Overview of the COVID-19 Outbreak on the Cruise Ship

**Figure 1:**
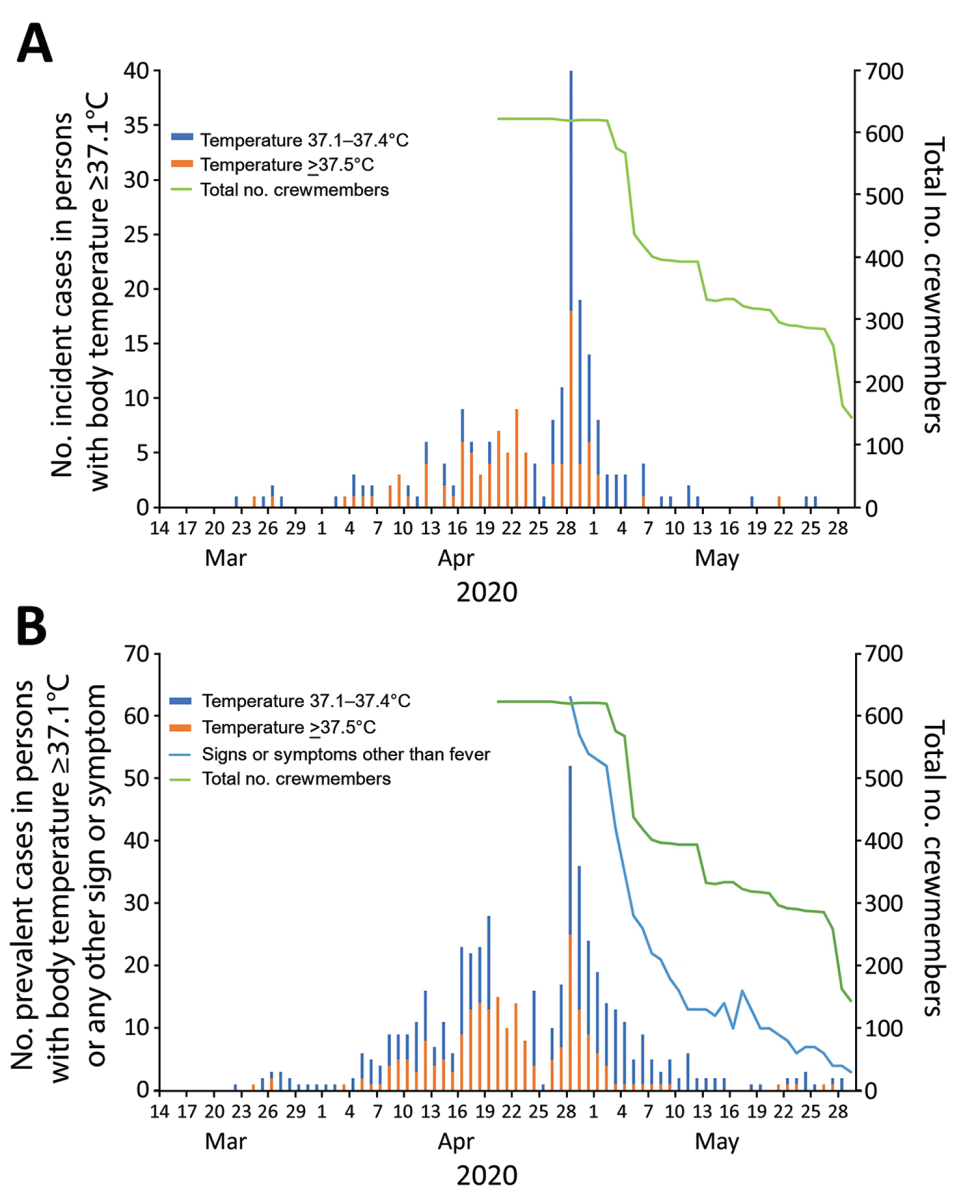
Number of incident cases of persons with body temperature >37.1°C and number of prevalent cases of persons with body temperature >37.1°C or any sign or symptom of coronavirus disease on a cruise ship, Nagasaki, Japan, March 14–May 29, 2020. A) Number of persons with illness onset, by date. Crewmembers started disembarking on May 3. B) Daily number of crewmembers who reported having a body temperature >37.1°C or coronavirus disease signs or symptoms. Signs or symptoms other than fever: cough, nasal congestion, sore throat, headache, olfactory dysfunction, taste disorder, conjunctival congestion, diarrhea, myalgia or arthralgia, fatigue, shortness of breath, and nausea or vomiting.

**Figure 2:**
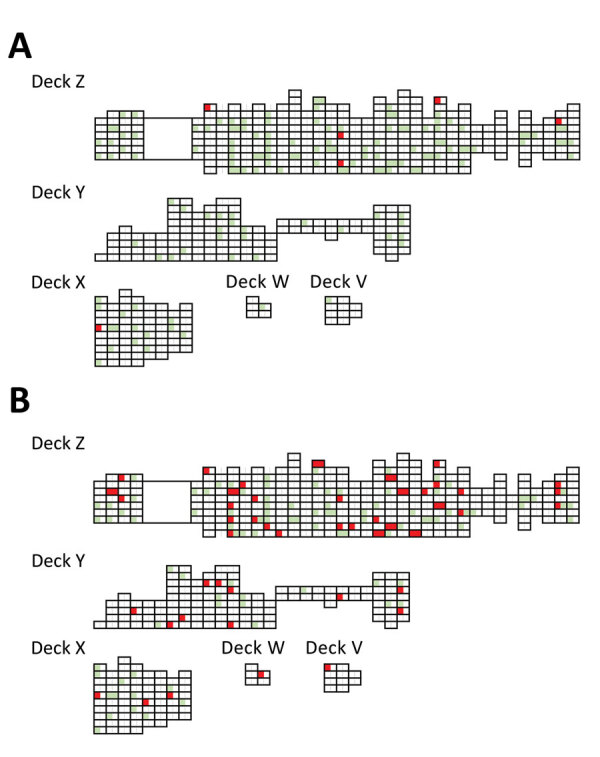
Initial cabin room locations of the crewmembers on cruise ship where a coronavirus disease outbreak occurred, Nagasaki, Japan, 2020. A) Red indicates distribution of crewmembers with onset of body temperature >37.1°C during March 22–27. Green indicates cabin rooms of crewmembers who did not have a body temperature >37.1°C. B) Red indicates distribution of crewmembers with onset of body temperature >37.1°C during March 22–April 19. Green indicates cabin rooms of crewmembers who did not have a body temperature >37.1°C.

A body temperature >37.1°C was first detected in a crewmember on March 22, and afterwards, 5 other crewmembers had a body temperature >37.1°C during March 24–27 (Figure 1, panel A). Their crew cabin rooms were not concentrated in a single area on the ship (Figure 2, panel A). However, all of these crew members belonged to the entertainment occupation group that boarded the cruise ship from several countries in Europe on March 18 and 19 (Appendix Figure 1). On April 2, another crewmember had a body temperature >37.1°C, and the number of persons with incident fever increased and peaked on April 28, decreasing thereafter (Figure 1, panel A). During March 22–May 29, a total of 211 (34%) had a body temperature >37.1°C. One crewmember who had a body temperature >37.1°C associated with cellulitis was excluded. Apart from the first wave of persons with a body temperature >37.1°C in late March, SARS-CoV-2 infection was distributed similarly across sex, and age group, nationality, and occupation type (Appendix Figure 1). The crew cabin rooms of crewmembers who had a body temperature >37.1°C also were widely distributed throughout the ship (Figure 2, panel B). No information on the ventilation system on the cruise ship was available. We compiled the daily number of crewmembers with a body temperature >37.1°C or with signs or symptoms; a peak occurred on April 28, after which the number gradually decreased until the end of May (Figure 1, panel B).

Among all 623 crewmembers, 149 cases were laboratory-confirmed and 107 probable case-patients who tested negative had clinical signs or symptoms indicative of COVID-19. Restricted to laboratory-confirmed cases, the attack rate for infection was 24%. When probable cases were included, the attack rate was 41%.

### Outbreak Control Measures and Management of Disembarkation

An emergency operations center was established in the prefecture office, and an onsite field response center was set up in the harbor near the cruise ship (*12*). Company staff stayed on board to communicate with both the ship and the public health authorities, and company staff introduced interventions to the ship. Online meetings among company staff on board, the onsite field response center, the emergency operations center, and Nagasaki University Hospital took place almost every morning. In the evening, online meeting between Japan’s Ministry of Health, Labour and Welfare and the emergency operations center took place. Through these communication and coordination mechanisms, we were able to share information, make informed decisions jointly, and implement interventions on the cruise ship.

Every nonessential worker had been separately isolated or quarantined in a single passenger cabin room and not allowed to leave his or her room since April 19. Essential workers who tested negative and had no signs or symptoms served meals or collected laundry for nonessential workers. When medically indicated, a nonessential worker was transported out of the room for medical care. To prevent secondary infection, several interventions were taken. Essential workers were provided with guidance and training for infection prevention on April 26. To ensure that the essential workers did not interact directly with isolated or quarantined crewmembers in their rooms, some interventions were put in place, such as distributing an individual thermometer to each crewmember on April 28 and streaming educational videos on COVID-19 infection prevention in the cabin rooms on April 29. To avoid missing the signs of disease progression and to be able to respond to critical patients in a timely manner, a field clinic was established, along with provision of a vehicle equipped with a computed tomography scanner and a medical transportation system. A remote health monitoring system was developed and introduced to support the cruise ship from outside of the ship (*17*). After the initial universal screening in April, only essential workers and medical personnel were reexamined when a sign or symptom indicative of COVID-19 was noted; reexaminations were limited in this way to prevent potential spread of the infection.

Starting May 3, those crewmembers who tested negative at universal screening were given priority to disembark; the ship’s medical doctor made the decision on the basis of the crewmember’s body temperature and signs or symptoms. Starting May 14, crewmembers who tested positive were able to disembark and travel back to their countries of origin, provided that they had a subsequent negative test result. Through this predisembarkation testing policy, a total of 495 crewmembers were able to disembark and leave Japan. On May 31, the cruise ship set sail for Manila, the Philippines, with the remaining 126 essential workers, none of whom had a positive test result.

### Clinical Outcomes

We compiled the clinical outcomes of all crewmembers and their signs or symptoms during their respective observation periods (Table 2). Among all crewmembers, 0.2% (1/623) had severe pneumonia, 0.3% (2/623) had moderate pneumonia, 32% (200/623) had mild illness, and 67% (420/623) had no signs or symptoms. Among crewmembers with laboratory-confirmed cases, 0.7% (1/149) had severe pneumonia, 1.3% (2/149) had moderate pneumonia, 62% (93/149) had mild illness, and 36% (53/149) had no signs or symptoms. Of the 11 crewmembers admitted to a hospital, 6 had COVID-19 pneumonia.

**Table 2:** Clinical outcomes of crewmembers on cruise ship where a coronavirus disease outbreak occurred, by SARS-CoV-2 test result, Nagasaki, Japan, 2020*

Clinical outcome†	All crewmembers	Test-positive	Test-negative
Total	623 (100)	149 (100)	474 (100)
Severe pneumonia	1 (0.2)	1 (0.7)	0
Moderate pneumonia	2 (0.3)	2 (1.3)	0
Mild illness	200 (32)	93 (62)	107 (23)
Asymptomatic	420 (67)	53 (36)	367 (77)
Fever (>37.5°C)	121 (19)	51 (34)	70 (15)
Symptom, n = 593‡			
Cough	45 (7.6)	32 (23)	13 (2.9)
Nasal congestion	34 (5.7)	23 (17)	11 (2.4)
Sore throat	32 (5.4)	22 (16)	10 (2.2)
Headache	32 (5.4)	18 (13)	14 (3.1)
Olfactory dysfunction	31 (5.2)	25 (18)	6 (1.3)
Taste disorder	28 (4.7)	23 (17)	5 (1.1)
Conjunctival congestion	24 (4.1)	13 (9.5)	11 (2.4)
Diarrhea	16 (2.7)	12 (8.8)	4 (0.9)
Myalgia or arthralgia	13 (2.2)	11 (8.0)	2 (0.4)
Fatigue	11 (1.9)	7 (5.1)	4 (0.9)
Shortness of breath	8 (1.4)	7 (5.1)	1 (0.2)
Nausea or vomiting	5 (0.8)	4 (2.9)	1 (0.2)

*Values are no. (%) except as indicated. SARS-CoV-2, severe acute respiratory coronavirus 2. †Severe pneumonia defined as illness requiring with intubation or intensive care unit admission; moderate pneumonia defined as illness requiring oxygen administration; mild illness defined as illness in patients who had coronavirus disease signs or symptoms without oxygen administration; asymptomatic, no clinical signs or symptoms. Body temperature data were obtained from the ship’s medical records and from the health monitoring system introduced by investigators on April 28, 2020. ‡Among 593 crewmembers who entered data into the health monitoring system introduced by investigators; 137 crewmembers tested positive and 456 tested negative for SARS-CoV-2.

### Clinical Course of Crewmembers Who Had Signs or Symptoms

During the observation period, 96 persons with laboratory-confirmed cases and 107 with probable cases experienced signs or symptoms. The median number of symptomatic days among laboratory-confirmed case-patients was 4 days (IQR 1–8 days) (Appendix Table). Among laboratory-confirmed case-patients who were asymptomatic at the time of universal screening, 58 (52%) subsequently experienced signs or symptoms. Among 38% of the symptomatic crewmembers, symptoms were intermittent, or additional COVID-19 signs or symptoms appeared sporadically over time (Appendix Figure 2).

## Discussion

We have described the key findings from the COVID-19 outbreak that occurred on a cruise ship with multinational crewmembers in Nagasaki City during April 20–May 29, 2020. Six crewmembers were hospitalized for COVID-19 pneumonia, 1 of whom had a severe case, but no deaths occurred. Our retrospective investigation revealed that the outbreak likely started in late March, with the infection introduced into this population from the entertainment occupation group that boarded the ship, which then spread widely inside the ship, irrespective of occupational group, nationality, or crew cabin room location, resulting in 149 laboratory-confirmed cases and 107 probable cases. Because transmission of SARS-CoV-2 from presymptomatic or asymptomatic patients is known to occur (*25*), certain social activities on the ship could have facilitated transmission which ultimately spread throughout the ship. The epidemic curve (Figure 1, panel A) shows that the number of incident case-patients with a body temperature >37.1°C peaked on April 28. The period between the universal implementation of the quarantine policy (April 19) and the peak of onset (April 28) was longer than the expected incubation period (*17*,*26*). This fact might be attributable to several reasons. For instance, before receiving training for infection prevention, essential workers might not have been able to sufficiently prevent infection transmission. Because an essential worker who was measuring body temperatures of isolated or quarantined crewmembers tested positive for SARS-CoV-2 on May 3, we speculate that infection could have spread through any interactions during those measurements or through sharing of the thermometers among the isolated or quarantined crewmembers. These factors might have contributed to further transmissions even after quarantine measures were enforced.

Management of the outbreak on Costa Atlantica was different from that observed on the Diamond Princess or other cruise ship outbreaks. The main difference was that the Costa Atlantica had only crewmembers whereas the Diamond Princess had both passengers and crewmembers. On Costa Atlantica, because passenger cabin rooms inside the ship were empty, crewmembers could be isolated or quarantined inside the ship, which was not possible on the Diamond Princess or other cruise ship outbreaks with passengers. For the outbreak on the Diamond Princess, priority testing was given to the high-risk population. Passengers with positive test results for SARS-CoV-2 were transported to medical facilities, and their clinical courses were followed. For those passengers without positive test results, a 14-day health observation period was set before disembarking (*27*,*28*). One study suggested the possibility that evacuating all on board early would have prevented many on the Diamond Princess from becoming infected (*29*). Early evacuation of all crewmembers was thus initially considered in the Costa Atlantica outbreak. However, there were not enough medical facilities or accommodations to isolate or quarantine all crewmembers in the city, and preparing other isolation facilities would have required installing sewage systems and using communal toilets, which could promote transmission, making such options both impractical and of questionable value. Because we regarded the area inside the ship as contaminated, we developed and introduced a health monitoring system (*13*), aiming to rapidly detect crewmembers requiring medical attention and to minimize the risk for secondary infection, which was an issue on the Diamond Princess (*13*,*30*). Debate is ongoing as to how to manage an outbreak of COVID-19 on a cruise ship, but we should take measures that are best suited for the particular context, especially in resource-limited situations.

As for clinical outcomes, we detected 3 crewmembers with moderate to severe pneumonia (2.0% of laboratory-confirmed cases and 1.2% of laboratory-confirmed and probable cases); this proportion was lower than that noted in a previous report in China, in which 14% of case-patients had severe illness and 5% had critical illness (*30*). However, the population on the Costa Atlantica included only crewmembers, who were considerably younger and healthier by selection (i.e., healthy worker effect).

Among the laboratory-confirmed cases, we determined 36% (42/149) to be in persons who were asymptomatic (*25*,*31*). In the Diamond Princess outbreak, the asymptomatic proportion was reported to be 55% (*4*), but after transfer to medical facilities, ≈20% of asymptomatic subjects had onset of signs or symptoms (*32*,*33*). We were able to follow the clinical courses of all laboratory-confirmed case-patients for >20 days, which prevented misclassification of presymptomatic cases as asymptomatic cases. We also obtained detailed clinical information after introducing the health monitoring system, which had a high usage rate, enabling individual crewmembers to report their signs or symptoms easily on a daily basis. These differences resulted in a lower proportion of asymptomatic cases in our study, which we think to be a more valid picture of the COVID-19 severity spectrum.

Of note, we did not repeat LAMP and PCR tests for SARS-CoV-2 for nonessential workers in our testing strategy. The main goal for our outbreak management was to prevent the spread of infection and to rapidly detect those persons who required medical attention (to provide them with appropriate and timely treatment); thus, repeat testing was not considered necessary as long as nonessential workers who were asymptomatic or had mild illness were isolated or quarantined. On the other hand, essential workers were repeatedly tested because of the potential to spread the infection. Before the ship’s departure, 107 probable cases were reported, which accounted for 23% of crewmembers with negative test results. With limited frequency of testing, some interval-censored infections might have been missed, but with a sensitive health monitoring system and an isolation and quarantine policy in place, we believe our operations were justifiable and effective.

Our study’s first limitation is that we might have underestimated the number of laboratory-confirmed cases because most crewmembers were only tested once. Second, the clinical signs or symptoms of the crewmembers before the introduction of the health monitoring system or after disembarkation could not be tracked, meaning additional symptomatic cases might have occurred.

In conclusion, we have described the epidemiology, along with our management approach, of a COVID-19 outbreak on a cruise ship with crewmembers isolated or quarantined inside the ship. Although SARS-CoV-2 can spread rapidly in closed settings, prompt isolation and quarantine and a sensitive surveillance system using a remote health monitoring approach could successfully control a COVID-19 outbreak on a cruise ship and result in timely medical care for affected persons.

Appendix Additional information about epidemiology of COVID-19 outbreak among crewmembers on cruise ship, Nagasaki City, Japan, April 2020.
