# Supplementary material for: Epidemiology of Coronavirus Disease Outbreak among Crewmembers on Cruise Ship, Nagasaki City, Japan, April 2020
Source: Emerg Infect Dis. 2021 Sep;27(9):2251–60. doi: 10.3201/eid2709.204596 (PMC8386778; doi:10.3201/eid2709.204596)
Supplement: Appendix — Additional information about epidemiology of COVID-19 outbreak among crewmembers on cruise ship, Nagasaki City, Japan, April 2020. [file 20-4596-Techapp-s1.pdf]

Article DOI: https://doi.org/10.3201/eid2709.204596

Epidemiology of Coronavirus Disease Outbreak among Crewmembers on Cruise Ship, Nagasaki City, Japan, April 2020

Appendix

Appendix Table. Number of days with symptoms/signs among crewmembers with positive test results for SARS-CoV-2*

Characteristic	No. persons	Median no. days symptomatic (interquartile range)
Any symptom, n = 149	96	4 (1–8)
Fever, ≥37.5°C, n = 149	51	2 (1–3)
Symptoms, n = 137		
Cough	32	4 (2–9.5)
Olfactory dysfunction	25	5 (2–8)
Nasal congestion	23	5 (1–13)
Taste disorder	23	2 (1–7)
Sore throat	22	3 (1–5)
Headache	18	3 (1–4)
Conjunctival congestion	13	3 (1–4)
Diarrhea	12	1.5 (1–3.5)
Myalgia or arthralgia	11	2 (1–8)
Fatigue	7	2 (1–3)
Shortness of breath	7	2 (1–2)
Nausea or vomiting	4	1 (1–3.5)

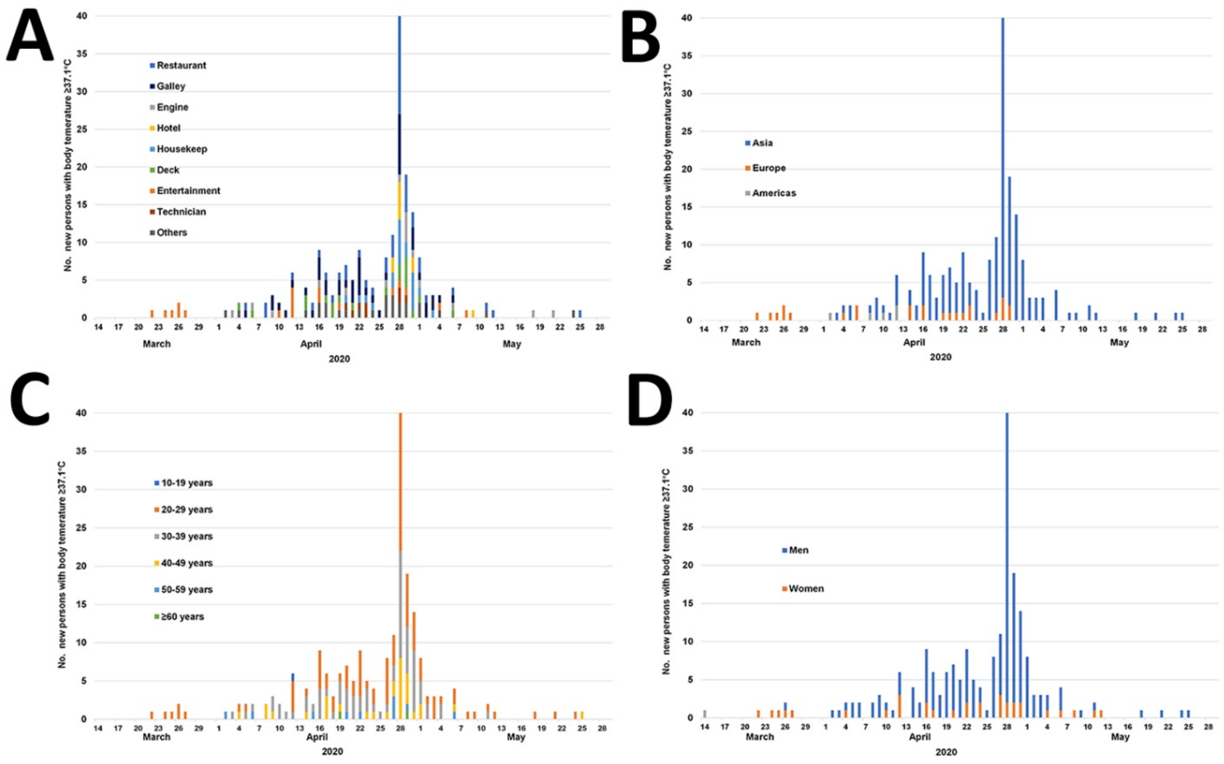

Appendix Figure 1. Number of persons with the onset of a body temperature ≥37.1°C on the respective date between March 14 and May 29, 2020. A) Categorized by occupation category. B) Categorized by region. C) Categorized by age group. D) Categorized by sex.

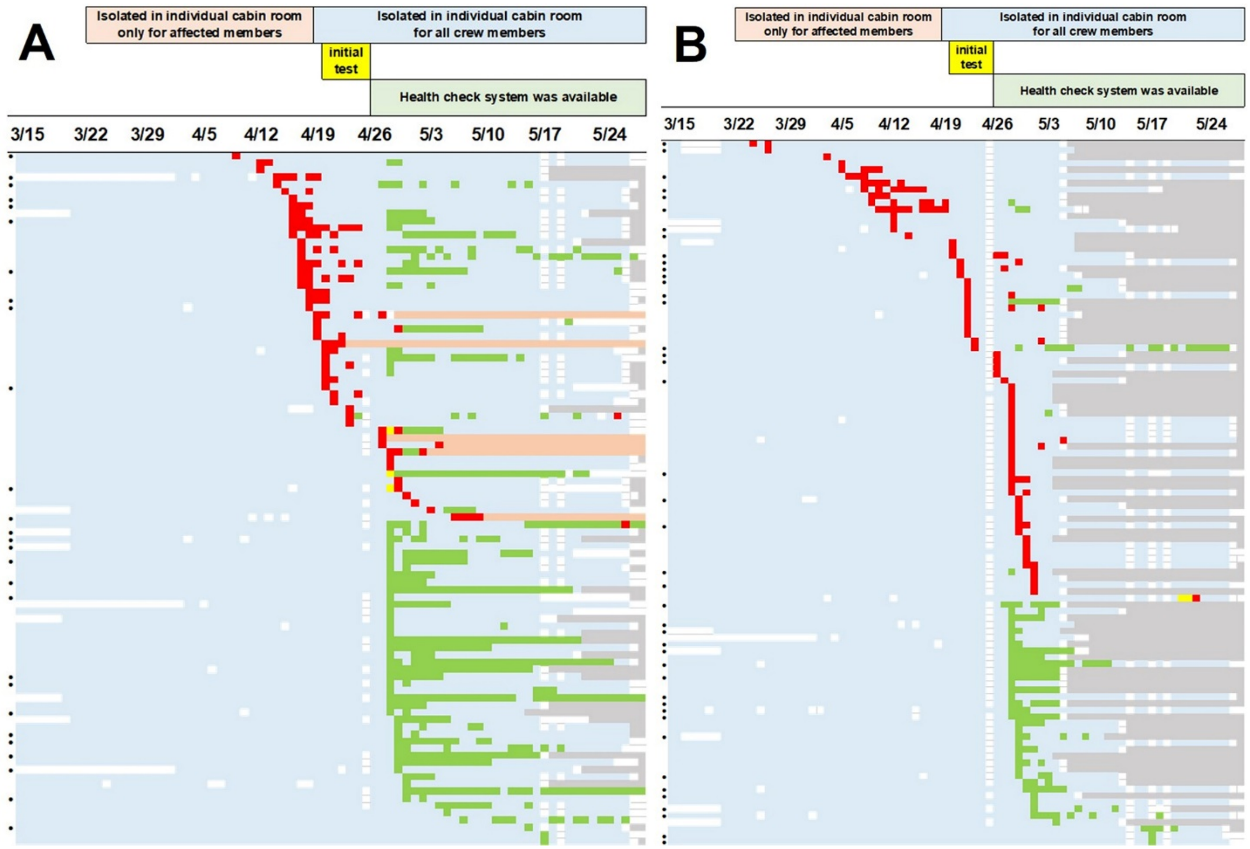

Appendix Figure 2. Individual clinical course of crewmembers who developed symptoms/signs during March 14 to May 29. A) Individual clinical course of crewmembers with positive test results for SARS-CoV-2 (n = 96). B) Individual clinical course of crewmembers with negative test results for SARS-CoV-2 (n = 107). Symptoms/signs included fever (≥37.5°C), cough, nasal congestion, sore throat, headache, olfactory dysfunction, taste disorder, conjunctival congestion, diarrhea, myalgia or arthralgia, fatigue, shortness of breath, and nausea or vomiting. Red indicates fever (≥37.5°C), and green indicates symptoms/signs other than fever. Yellow indicates a person who had both fever and some other symptoms/signs. Blue denotes having no symptoms, gray denotes disembarkation to depart Japan (cleared based on clinical and laboratory results), and orange denotes disembarkation for hospital admission (transported to hospital for medical care). A black circle on the far left shows a crewmember with a risk factor for severe COVID-19: underlying disease (hypertension, diabetes, cardiovascular disease, or asthma), history of smoking, or obesity (BMI ≥30).
